# The *NPC1L1* Gene Exerts a Notable Impact on the Reduction of Low-Density Lipoprotein Cholesterol in Response to Hyzetimibe: A Factorial-Designed Clinical Trial

**DOI:** 10.3389/fphar.2022.755469

**Published:** 2022-03-11

**Authors:** Jianwei Liao, Liyun Yang, Luping Zhou, Hongbin Zhao, Xiao Qi, Yimin Cui, Dongsheng Ouyang

**Affiliations:** ^1^ Department of Clinical Pharmacology, Xiangya Hospital, Central South University, Changsha, China; ^2^ Institute of Clinical Pharmacology, Central South University, Changsha, China; ^3^ Zhejiang Hisun Pharmaceutical Co. Ltd, Taizhou, China; ^4^ Hunan Key Laboratory for Bioanalysis of Complex Matrix Samples, Changsha, China; ^5^ Peking University First Hospital, Beijing, China

**Keywords:** hyzetimibe, ezetimibe, *NPC1L1* gene, factorial design, SNP, precise treatment

## Abstract

**Background:** Hyzetimibe is a novel inhibitor of cholesterol that specifically targets the *NPC1L1* gene. Significant inter-individual variability suggests the existence of an abundance of poor responders and non-responders. In addition, the current literature is inconsistent and controversial regarding the potential impact of the Niemann-Pick C1-Like 1 (NPC1L1) gene on low-density lipoprotein cholesterol (LDL-C) reduction. In light of these concerns, we performed a high-quality clinical trial to investigate the specific characteristics of *NPC1L1* gene variation on LDL-C reduction.

**Methods:** This was a multicenter, randomized, double-blind, placebo-controlled, clinical trial with a factorial design. Qualified patients were randomly assigned to one of six treatments: placebo, hyzetimibe (10 or 20 mg), atorvastatin, and atorvastatin plus hyzetimibe (10 or 20 mg). Fasting blood samples were collected and genotyped, and the concentrations of LDL-C and the targeted drug trough were determined to investigate the association between the *NPC1L1* gene expression and the reduction of LDL-C.

**Results:** In total, 727 individuals were initially recruited; of these, 444 were eligible to begin the trial. We identified one SNP (g1679C > G) that exerted significantly different impacts on LDL-C levels. As monotherapy, CC carriers experienced significantly higher reductions in the mean LDL-C (−23.99%) than either the GG (−16.45%, *p* < 0.01) or GC (−13.02%, *p* < 0.01) carriers in the hyzetimibe (20 mg) group. In contrast, when co-administered with atorvastatin, GC carriers experienced greater LDL-C reduction than non-GC carriers (-52.23% vs. −45.03%) in the hyzetimibe (20 mg) plus atorvastatin group. Furthermore, the proportions of individuals experiencing a reduction in LDL-C by >50% increased as the dose of hyzetimibe increased from 16.1% to 65.4%.

**Conclusion:** The g1679C > G SNP in the *NPC1L1* gene is critical and exerts a differential impact on the response to hyzetimibe treatment. Heterozygotic patients respond with poor efficacy when treated by monotherapy but show good responses in terms of LDL-C reduction when hyzetimibe was co-administered with atorvastatin. To treat hypercholesterolemia in a precise manner with hyzetimibe, it is necessary to identify genotype patients for the g1679C > G SNP. We also highlight the potential necessity for identifying the appropriate subjects to be treated with ezetimibe.

Clinical Trial Registration: [https://clinicaltrials.gov/], identifier [CTR20150351]

## Introduction

The treatment of dyslipidemia has become critical for the early prevention and treatment of cardiovascular diseases, and the reduction of low-density lipoprotein cholesterol (LDL-C) plays a critical role in reducing the risk of cardiovascular events in both primary and secondary prevention (Cholesterol Treatment Trialists' Collaborators, et al., 2012).

Hyzetimibe (HS-25) is the second inhibitor of cholesterol absorption to be developed in China and was approved for marketing by the National Medical Products Administration of China (NMPA) in June 2021. A pharmacokinetic study of oral ^14^C-radiolabeled hyzetimibe revealed that a change in the hydroxyl group within the drug structure made it easier for hyzetimibe to combine with glucuronic acid, thus leading to the increased urinary excretion of hyzetimibe than ezetimibe (Liao et al., 2021). Hyzetimibe only results in a modest reduction in LDL-C and acts by inhibiting the intestinal cholesterol transporter Niemann-Pick C1-Like 1 (*NPC1L1*) (Ruan et al., 2014), which plays a critical role in the intestinal absorption of cholesterol (Altmann et al., 2004). Data obtained from clinical trials showed that hyzetimibe monotherapy reduced the levels of LDL-C by 15–18%. However, a significant variation was noted in the response to hyzetimibe, thus suggesting the existence of patients who show a poor response to hyzetimibe treatment or do not respond at all. As *NPC1L1* is the molecular target of hyzetimibe, it follows that the expression levels of *NPC1L1* reflect the efficiency of cholesterol absorption (Hui et al., 2008). Therefore, the response in variability may be partly attributed to the presence of the inherited genetic variation in *NPC1L1*.

Ezetimibe is another inhibitor of cholesterol absorption and was first approved for marketing by the Food and Drug Administration (FDA) in 2002 (Dujovne et al., 2002; Knopp et al., 2003)*.* Ezetimibe monotherapy has shown to reduce the levels of LDL-C by 15–20%; an additional reduction of −13% to −20% could be attained when ezetimibe was combined with statin therapy, depending on the concentration of statin administered (Davidson et al., 2002; Ballantyne et al., 2003).

Pharmacogenomic studies were carried out in over 1,000 patients with hypercholesterolemia to investigate the impact of polymorphisms in the *NPC1L1* gene on LDL-C reduction in response to ezetimibe (Simon et al., 2005). However, results were inconsistent, and even controversial; potentially due to the study design. The patients involved in this study had hypercholesterolemia and had been recruited on the ezetimibe add-on to statin for effectiveness (EASE) clinical trial and the Vytorin *versus* atorvastatin (VYVA) clinical trial. The EASE study was designed to evaluate the effects of ezetimibe, in combination with a stable regimen of statin therapy (any dose, any brand). In contrast, the VYVA study aimed to investigate the effects of a combination of ezetimibe and simvastatin in comparison with atorvastatin in patients with hypercholesterolemia.

These trials involved only a single dose of ezetimibe; this made it difficult to identify the specific association between the drug dose and the response exerted by the genetic variation in the *NPC1L1* gene. Furthermore, neither of these studies considered ezetimibe monotherapy; this made it difficult to investigate the specific response to ezetimibe monotherapy. In addition, in the EASE cohort, ezetimibe was combined with statins (any dose and any brand); differential negative feedback mechanisms arising from different statins and doses, along with the complex background of statin therapy, made it difficult to determine the precise effects of these treatments.

Pisciotta et al. (2007) were the first to reveal the effect of the combination of ezetimibe and statins in genotype-confirmed heterozygous monotherapy in Caucasian familial hypercholesterolemia. Analysis showed that LDL-C was reduced by −18.9 ± 1.0% and −23.3 ± 2.3% (*n* = 65, *p* < 0.07) in Caucasian familial hypercholesterolemia and by −29.2 ± 1.0% and −33.7 ± 2.6% (*n* = 50, *p* < 0.06) in patients with primary hypercholesterolemia with the CC genotype and non-CC genotype of the g1679C > G SNP, respectively.

Subsequently, the Myocardial Infarction Genetics Consortium Investigators et al. (2014) found that heterozygous carriers of inactivating *NPC1L1* mutations had a 53% relative risk reduction for coronary heart disease and had lower levels of LDL-C than non-carriers (*p* = 0.04).

Schweitzer et al. (2015) were the first to characterize the *NPC1L1* gene and proteome in an exceptional responder to ezetimibe and attempted to identify genetic variations that caused functional changes. The combination of L52P-NPC1L1 and I300T-NPC1L1 amino acid changes caused a functional reduction in cholesterol uptake in the presence of ezetimibe, which was considered as a key aspect of the exceptional response.

Clearly, there is a lack of specific data relating to the potential impact of gene variations in *NPC1L1* on the effect of cholesterol absorption inhibitors; existing data are inconsistent and unclear. Consequently, there is a critical need to carry out a high-quality clinical trial to investigate the characteristics of gene variation with exceptional responders or poor responders to drug treatment. Thus, we carried out a multicenter, randomized, double-blind, balanced-parallel, and placebo-controlled trial in 727 Chinese patients with primary hypercholesterolemia. For each patient, we characterized variability in the DNA sequence of the *NPC1L1* gene and subsequently investigated the association between *NPC1L1* and LDL-C reduction induced by hyzetimibe treatment.

## Methods

### Patients

The trial was carried out between 2015 and 2019, and patients with primary hypercholesterolemia from 43 medical institutions across China were enrolled. The inclusion criteria were as follows: individuals aged 18–70 years with primary hypercholesterolemia, defined as a calculated LDL-C of 3.36–4.88 mmol/L, following dietary control for >4 weeks; individuals in which the difference between two LDL-C concentrations, measured more than 1 week apart during the washout period, was less than 12%; and individuals who had not received any cholesterol-lowering drugs, such as statins, for 6 weeks prior to the trial. All patients provided written and informed consent before enrollment.

The exclusion criteria were as follows: homozygous familial hypercholesterolemia; severe heart, liver, lung, kidney, and other important organ diseases; serious muscle abnormalities and neuropathies; uncontrolled endocrine disease known to influence serum lipids or lipoproteins; ezetimibe intolerance; patients who were diabetic and aged >40 years; diabetes with cardiovascular risks; blood pressure ≥160/100 mmHg; thyroid dysfunction; a history of malignant tumors; triglyceride ≥3.99 mmol/L (350 mg/dl); known impairment of renal function; active or chronic hepatic or hepatobiliary disease; atherosclerotic cardiovascular disease; uncontrolled cardiac arrhythmia; and HCV or HBsAg positive. We also excluded patients who were taking fibric chemicals such as fenofibrate, gemfibrozil, probucol, warfarin, corticosteroids, cyclosporine, or other immunosuppressives within 12 weeks prior to the trial.

The entire trial was conducted in accordance with the principles of the Declaration of Helsinki for biomedical research involving human subjects and according to the International Conference on Harmonization Guidelines for Good Clinical Practice.

### Trial Design

A multicenter, randomized, double-blind, balanced-parallel, and placebo-controlled group trial was designed carefully and carried out in a strict manner. The protocol was reviewed and approved by the Independent Ethics Committee of the Second Affiliated Hospital Zhejiang University School of Medicine and other participating medical institutions. The clinical trial was approved by the National Medical Products Administration of China (Approval number: 2014L00799). The clinical trial was registered on the Chinese Clinical Trial Registration and Information Platform (Registration number: CTR20150351).

On the first day of informed consent, patients received dietary counseling and were randomly assigned by a centrally managed service (the Interactive Web Response System) to receive drug treatment according to randomized stratification by site, age, sex, and baseline LDL-C level. The 4-week screening phase included the washout of previous lipid-modifying drug therapy and was instructed to follow a stricter diet throughout the trial. At week 0, patients were randomly assigned to one of six treatments for 12 weeks: placebo (PLB), hyzetimibe 10 mg (HS25-10 mg), hyzetimibe 20 mg (HS25-20 mg), atorvastatin 10 mg (ATO), atorvastatin (10 mg) plus hyzetimibe 10 mg (ATO + HS25-10 mg), and atorvastatin (10 mg) plus hyzetimibe 20 mg (ATO + HS25-20 mg).

### Measurement of Serum Lipids

Fasting blood samples were collected at the baseline and weeks 2, 4, 8, and 12. LDL-C levels were measured by using an AU5800 automatic Beckman biochemical analyzer (Beckman Coulter) at the central laboratory.

### Measurement of Plasma Drug Concentrations

Plasma concentrations of hyzetimibe, hyzetimibe-M1 (HS-25 M1, the main metabolite of hyzetimibe), along with atorvastatin trough concentrations, were determined by liquid chromatography (Shimadzu) and mass spectrometry (AB SCIEX) at the central laboratory.

### DNA Sequencing

DNA samples were sequenced by next-generation sequencing performed by BGI (Beijing, China). The coding genes for the target NPC1L1 protein were sequenced, including the exons, intron junctions, 3′-untranslated region (3′- UTR), 5′-UTR, and the promoter.

### Statistical Analysis

The percentage reduction of the LDL-C level from the baseline to final assessment was considered as the primary efficacy end point and was analyzed by a two-way analysis of variance (ANOVA) model that extracted effects due to placebo and treatment (hyzetimibe 10 and 20 mg). Next, we performed a series of data comparisons (pooled atorvastatin plus hyzetimibe (10 mg, 20 mg)) *versus* hyzetimibe ((10 mg, 20 mg) or atorvastatin). Statistical analyses were performed by SAS software (version 9.4, SAS Institute Inc., United States). A *p* value less than or equal to 0.05 was considered statistically significant.

## Results

**FIGURE 1:**
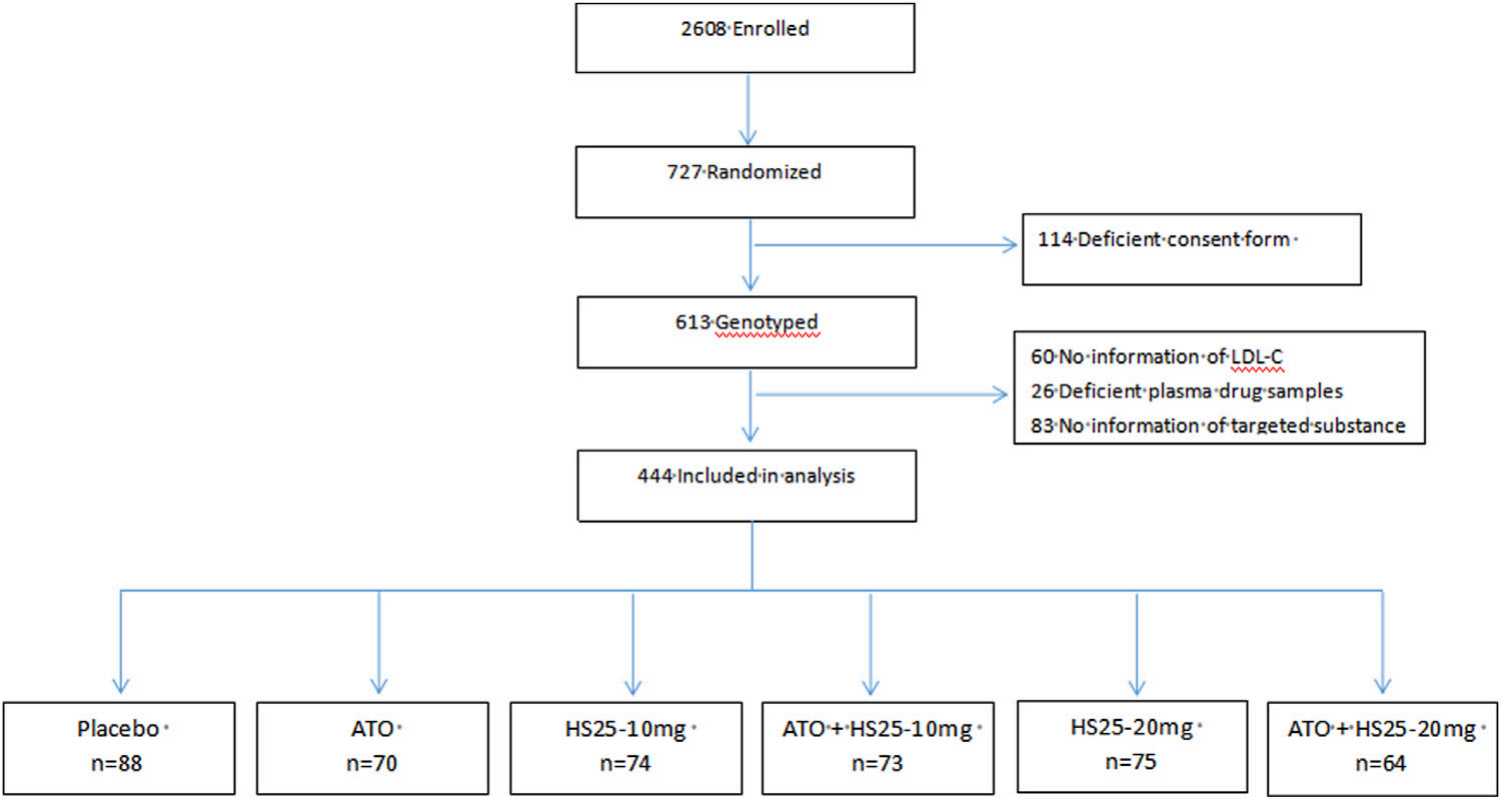
Trial profile. ATO: atorvastatin, HS25: hyzetimibe.

Overall, 2,608 individuals were enrolled initially; of these, 727 were randomly assigned, and 444 were finally recruited for the analysis of genetic variation in *NPC1L1*; patients were excluded due to incomplete data relating to C trough and LDL-C, non-target substances in the plasma, or the absence of genotyping data (Figure 1). The treatment groups were well balanced with regards to demographics and baseline characteristics (Supplementary Table S1).

Table 1 shows the genotype frequency of the minimum allele frequency (MAF) of the four most common SNPs in the *NPC1L1* gene (rs374773929, rs4720470, rs2301935, and rs2072183); the MAFs for these SNPs all exceeded 10% in Chinese patients with primary hypercholesterolemia. However, only those with the g1679C > G (rs2072183) genotype were associated with the lipid-lowering effect of hyzetimibe. The frequency of the g1679C > G genotype was similar to that in a previous Japanese population (*p* > 0.05) but was statistically different from the frequency recorded in a healthy population of Chinese and Japanese patients (*p* < 0.001), as well as in Canadian patients with primary hypercholesterolemia (*p* < 0.001). These findings indicated that there was a significant difference in the frequency of the g1679C > G variant when compared between healthy Chinese subjects and those with dyslipidemia; there was a clear genetic variation between Chinese and Canadian patients with dyslipidemia (Table 2) (Simon et al., 2005; Kashiwabara et al., 2014; Lou et al., 2015).

**TABLE 1:** Genotype frequency of SNP with the minor allele frequency (MAF) more than 10%.

SNP/db SNP	Genotype	No	Frequency	Allele	Frequency
rs374773929	0/0	1	0.2%	0	50.1%
	0/1	250	40.8%	1	20.4%
	0/2	362	59.1%	2	29.5%
	Total	613	100.0%	—	100.0%
rs4720470	CC	273	44.5%	—	—
	CT	276	45.0%	C	67.0%
	TT	64	10.4%	T	33.0%
	Total	613	100.0%	—	100.0%
rs2301935	TT	203	33.1%	—	—
	TG	284	46.3%	T	56.3%
	GG	126	20.6%	G	43.7%
	Total	613	100.0%	—	100.0%
rs2072183	GG	253	41.3%	—	—
	GC	279	45.5%	G	64.0%
	CC	81	13.2%	C	36.0%
	Total	613	100.0%	—	100.0%

**TABLE 2:** Genotype frequency of g1679C > G (SNP rs2072183) in this study and in the literature.

Genotype	Primary hypercholesterolemia, Chinese *Han* (this study) N (%)	Healthy	Primary hypercholesterolemia, Japanese N (%)	Healthy	Primary hypercholesterolemia, Canadian N (%)
		Chinese *Han*		Japanese	
		*N* (%)		*N* (%)	
GG	253 (41%)	53 (13%)	41 (36%)	27 (19%)	5 (5%)
GC	279 (46%)	208 (49%)	57 (49%)	46 (56%)	40 (40%)
CC	81 (13%)	163 (38%)	17 (15%)	50 (35%)	56 (55%)
*p*	**—**	<0.0001	0.5276	<0.0001	<0.0001

**FIGURE 2:**
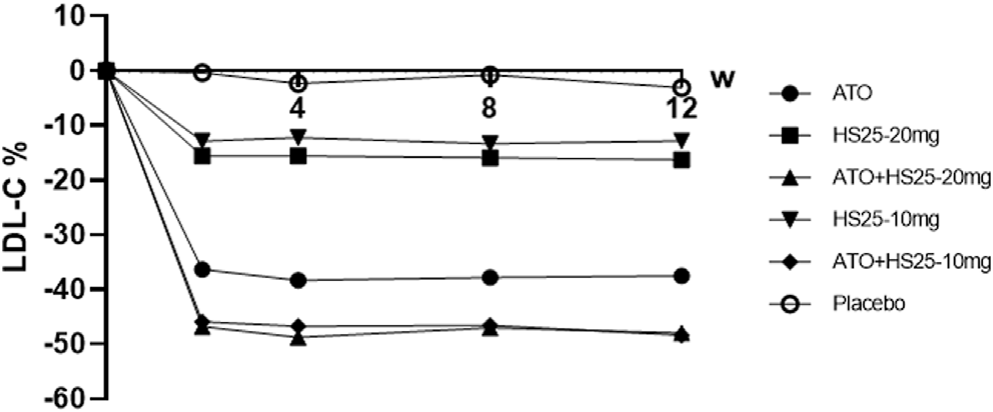
Changes of LDL-C at the study time points compared to baseline values. LDL-C: low-density lipoprotein cholesterol, ATO: atorvastatin, HS25: hyzetimibe.

Treatment with hyzetimibe (10 and 20 mg) resulted in a mean percentage reduction from the baseline to end point in the plasma concentration of LDL cholesterol (12.82% and 16.29%, respectively) as compared to a decrease of only 3.12% in the placebo group (*p* < 0.01). The reduction of LDL cholesterol by hyzetimibe occurred early (2 weeks) and was maintained throughout the 12-week treatment period. The coadministration of hyzetimibe and atorvastatin resulted in a significantly greater mean reduction of LDL-C than either atorvastatin alone or hyzetimibe alone (Figure 2; Supplementary Table S2).

### The Effect of Genotype on Monotherapy

**FIGURE 3:**
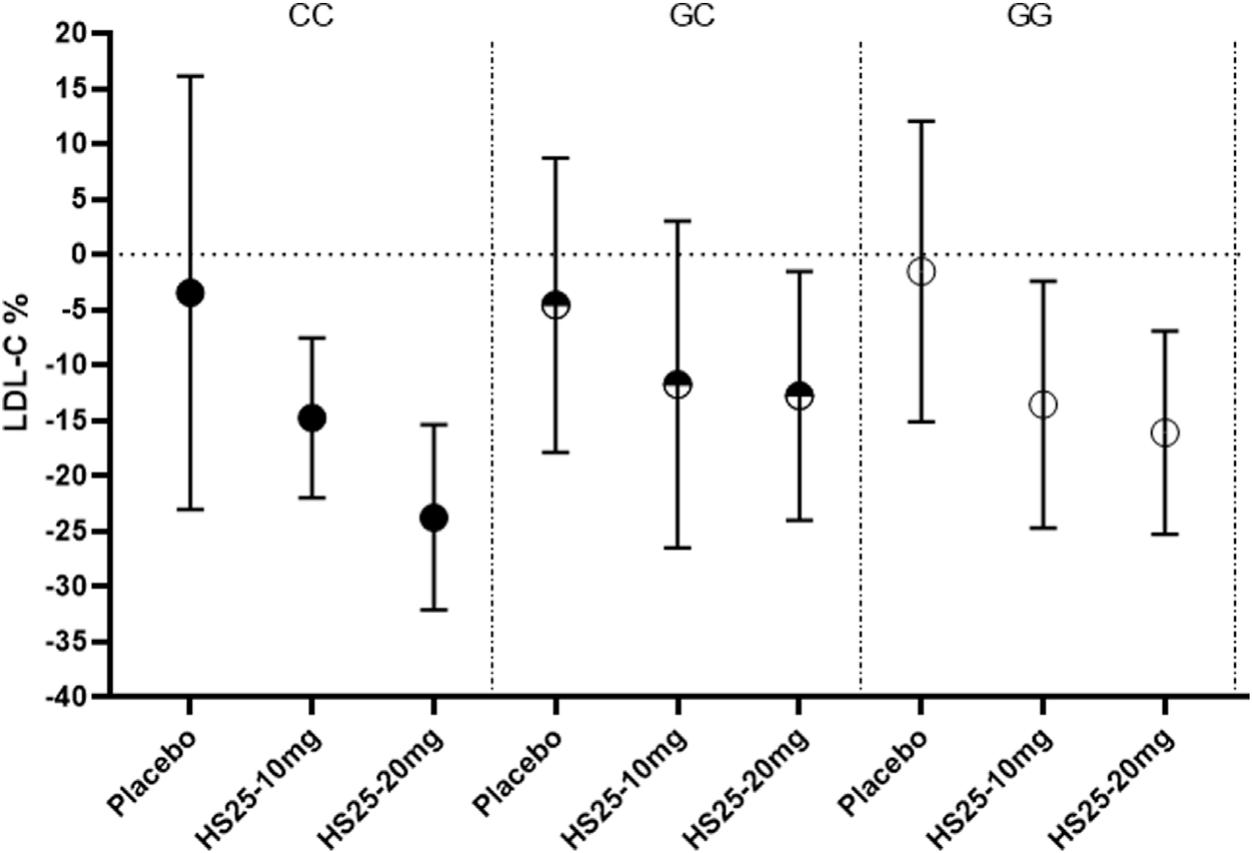
Changes of LDL-C in patients with the three genotypes of the g1679C > G SNP in the monotherapy group. LDL-C: low-density lipoprotein cholesterol, HS25: hyzetimibe.

Next, we investigated the relationship between the genetic variation of g1679C > G and the serum lipid profile. The relative reduction of LDL-C levels across the three genotypes in each treatment group is presented in Figure 3. No association was observed between single-nucleotide polymorphism and the change in LDL-C levels either in the placebo or atorvastatin pooled treatment groups. However, there were significant differences among the three genotypes in terms of hyzetimibe (20 mg) treatment. The reduction in LDL-C levels in the three genotypes were as follows: CC (23.99%) > GG (16.45%) > GC (13.02%) (Table 3). When the effects of the placebo were further deducted, the LDL-C reductions exhibited a rank order of CC (−20.49%) > GG (14.67%) >GC (−8.46%) (Table 3), and the absolute reduction was 0.96 mmol/L [95% confidence interval CI): 0.68–1.24] for the CC genotype, 0.52 mmol/L (95% CI: 0.30–0.73) for the GC genotype, and 0.65 mmol/L (95% CI: 0.44–0.86) for the GG genotype, respectively (Table 4).

**TABLE 3:** Least-squares mean percentage changes from the baseline to final assessment in LDL-C variables.

	Mean ± SEM(n)			Difference*		Difference**	
	Placebo	HS25-10 mg	HS25-20 mg	Mean ± SEM (95%CI)	p	Mean ± SEM	p
CC	−3.50 ± 8.07 (10)	−14.45 ± 5.20 (9)	−23.99 ± 3.40 (16)	−10.95 ± 3.16 (−17.61,−4.29)	0.0029	−20.49 ± 2.27 (−15.81,−25.17)	<0.0001
GC	−4.56 ± 3.03 (39)	−12.14 ± 2.59 (37)	−13.02 ± 2.65 (33)	−7.58 ± 0.65 (−8.87,-6.23)	<0.0001	−8.46 ± 0.68 (−9.81,−7.11)	<0.0001
GG	−1.78 ± 2.95 (39)	−13.78 ± 2.80 (28)	−16.45 ± 2.67 (26)	−12.00 ± 0.72 (−13.43,−10.57)	<0.001	−14.67 ± 0.72 (−6.11,−13.23)	<0.0001

*HS25-10 mg (pooled) vs. placebo (pooled). **HS25-20 mg (pooled) vs. placebo (pooled). Reduction rate of LDL-C= (LDL-C, in week 12—LDL-C in baseline)/LDL-C in baseline. Abbreviations: LS, least squares; SEM, standard error; CI, confidence interval. LS-Mean estimates were based on the pairwise comparisons from an ANOVA model with fixed effects for gender and with the baseline LDL-C value as a covariate. The *p* values are from F-tests from the ANOVA model.

**TABLE 4:** Absolute LDL-C reduction of three genotypes compared to the baseline value.

	CC (mmol/L, 95%CI)	GC (mmol/L, 95%CI)	GG (mmol/L, 95%CI)	*p*
Placebo	−0.14 (0.52,−0.80)	−0.18 (0.06,−0.42)	−0.07 (−0.16,−0.30)	0.0062
HS25-10 mg	−0.57 (−0.13,−1.00)	−0.48 (−0.28,−0.69)	−0.54 (−0.32,−0.76)	0.0480
HS25-20 mg	−0.96 (−0.68,−1.24)	−0.52 (−0.30,−0.73)	−0.65 (−0.44,−0.86)	<0.0001

*ANOVA test. CI, confidence interval.

**FIGURE 4:**
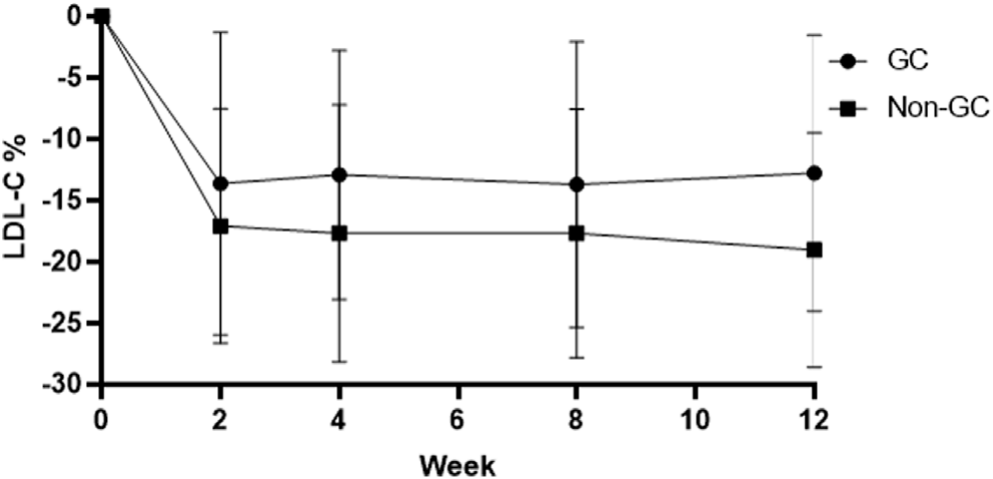
Changes of LDL-C in patients with the GC genotype and the non-GC genotype in the hyzetimibe 20 mg treatment group each week. LDL-C: low-density lipoprotein cholesterol.

Patients with a homozygous genotype (CC and GG) in the hyzetimibe (20 mg) group showed similar reductions in the levels of LDL-C reduction at 0.77 mmol/L (95% CI: 0.60–0.94, −19.34%, Figure 4). Data also showed that patients with the CC genotype exhibited significantly greater reductions in LDL-C levels than ezetimibe, thus indicating a greater clinical benefit.

Furthermore, there is a significant correlation between LDL-C levels and the administration of hyzetimibe in patients with the g1679C > G SNP and the CC genotype but not in those with the GC and GG genotypes. The correlation coefficient between LDL-C levels and hyzetimibe reached 0.998, and higher doses were associated with better effects.

### The Effect of Genotype on Combination Therapy

**FIGURE 5:**
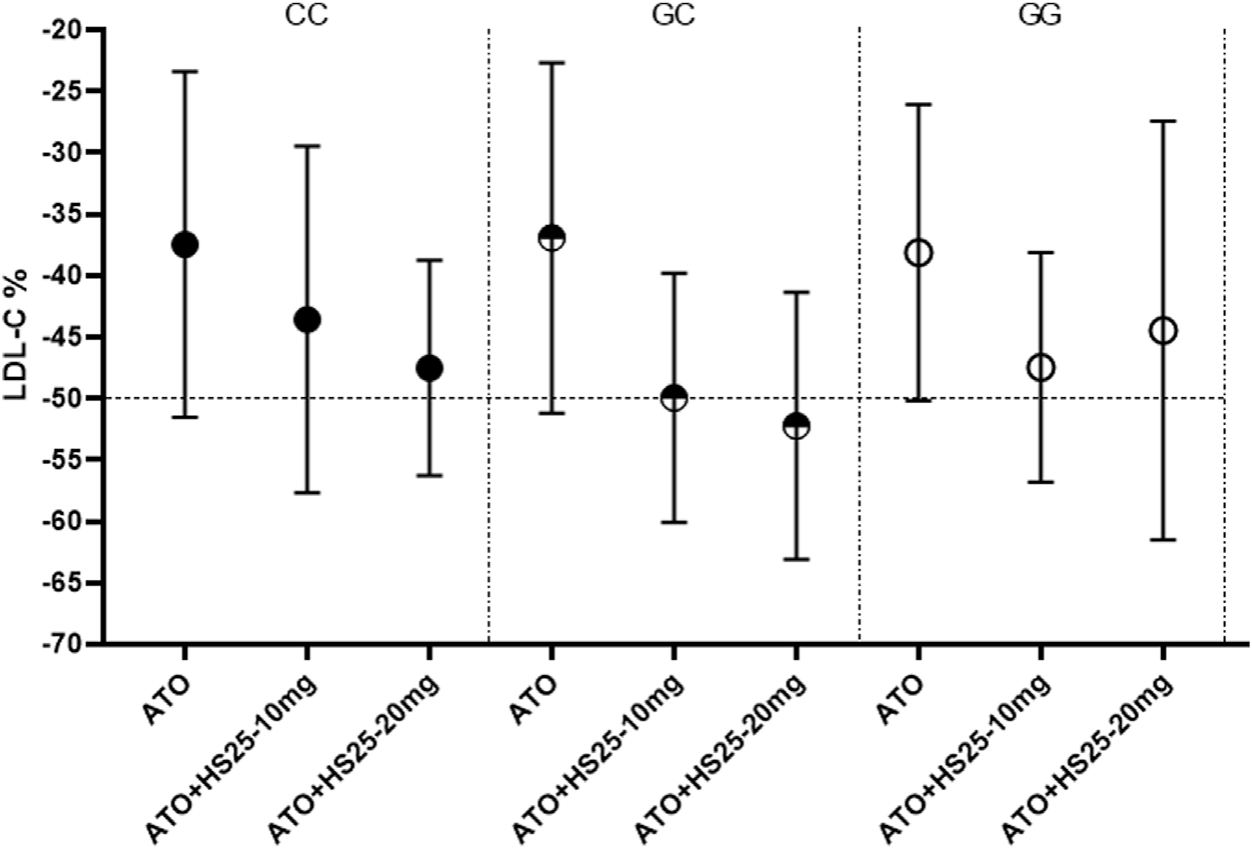
Changes of LDL-C to the three genotypes of SNP g1679C > G in the combining treatment group. LDL-C: low-density lipoprotein cholesterol, ATO: atorvastatin, HS25: hyzetimibe.

**FIGURE 6:**
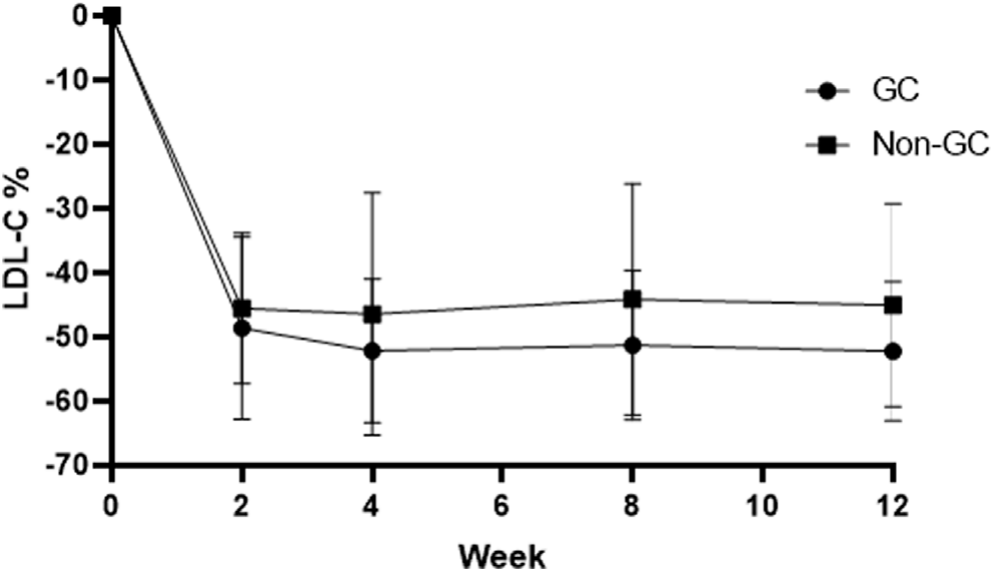
Changes of LDL-C in the GC genotype and non-GC genotype in the atorvastatin plus hyzetimibe 20 mg treatment group each week. LDL-C: low-density lipoprotein cholesterol.

No significant differences were observed between LDL-C reduction and the g1679C > G genotypes irrespective of whether we considered atorvastatin plus 10 mg hyzetimibe or atorvastatin plus 20 mg hyzetimibe. However, patients with a heterozygous genotype (the GC genotype) exhibited a greater reduction in the median level of LDL-C than the non-GC genotypes (−52.23% *versus* −45.03%, *p <* 0.05) in the hyzetimibe (20 mg) plus atorvastatin group (Figure 5; Table 5). Furthermore, we observed significant differences when we performed combined analysis on the three genotypes (*p* = 0.051) with respect to the reduction of LDL-C; patients exhibiting the GC genotype exhibited a greater reduction in LDL-C than those with the GG genotype (−50.87% *versus* −45.84%, *p <* 0.05, Figure 6).

**TABLE 5:** Least-squares mean percentage changes in LDL-C variables with hyzetimibe plus atorvastatin.

	Mean ± SEM(n)			Difference*		Difference**	
	Ato	Ato/HS25-10 mg	Ato/HS25-20 mg	Mean ± SEM (95%CI)	p	Mean ± SEM (95%CI)	p
CC	−36.77 ± 6.37 (11)	−44.22 ± 6.13 (8)	−47.47 ± 4.10 (7)	−7.45 ± 2.91 (−13.60,−1.30)	0.0204	−10.70 ± 2.72 (−16.47, −4.93)	0.0012
GC	−37.42 ± 2.83 (31)	−49.82 ± 2.27 (39)	−52.33 ± 3.05 (26)	−12.40 ± 0.61 (−13.62,−11.18)	<0.0001	−14.91 ± 0.78 (−16.47, −13.35)	<0.0001
GG	−38.17 ± 2.85 (28)	−47.17 ± 3.19 (26)	−44.65 ± 3.37(31)	−9.00 ± 0.82 (−10.65,−7.35)	<0.0001	−6.48 ± 0.82 (−8.12, −4.84)	<0.0001

*Atorvastatin + HS25-10 mg (pooled) vs. atorvastatin (pooled). **Atorvastatin + HS25-20 mg (pooled) vs. atorvastatin (pooled). Reduction rate of LDL-C= (LDL-C, in week 12—LDL-C, in baseline)/LDL-C, in baseline. Abbreviations: LS, least squares; SEM, standard error; CI, confidence interval. LS-Mean estimates were based on the pairwise comparisons from an ANOVA model with fixed effects for gender and with the baseline LDL-C value as a covariate. The *p* values are from F-tests from the ANOVA model.

Patients with the GC genotype who received a combination of atorvastatin plus 20 mg hyzetimibe exhibited a significantly higher reduction in LDL-C (14.91%) than those with the CC (10.71%) or GG (6.48%) genotypes when compared to atorvastatin alone (Table 5). Similar results were also observed for patients who were treated with atorvastatin plus 10 mg hyzetimibe. Furthermore, increased doses of hyzetimibe were associated with greater reductions in LDL-C reduction (by more than 50%) in the following rank order: hyzetimibe (20 mg) plus atorvastatin (65.4%) > hyzetimibe (10 mg) plus atorvastatin (46.2%) > atorvastatin alone (16.1%), *p* < 0.001 (Table 6).

**TABLE 6:** Meeting rate of LDL-C reduction by >50% in the hyzetimibe plus atorvastatin group.

	Ato (%,n)	Ato + HS25-10 mg (%,n)	Ato + HS25-20 mg (%,n)	*p*
CC	18.2% (2/11)	37.5% (3/8)	28.6% (2/7)	0.640
GC	16.1% (5/31)	46.2% (18/39)	65.4% (17/26)	0.0007
GG	10.7% (3/28)	46.2% (12/26)	45.2% (14/31)	0.006

ANCOVA test.

## Discussion

This is the first randomized clinical trial (based on a factorial design with six arms) to demonstrate an association between the *NPC1L1* gene and the reduction of LDL-C levels in response to drug treatment with an inhibitor of cholesterol absorption. Our investigation revealed that genetic variations in *NPC1L1* exert notable impacts on the extent of LDL-C reduction in response to hyzetimibe treatment in patients with primary hypercholesterolemia.

Among the common NPC1L1 haplotypes, only the g1679C > G SNP was found to exhibit a significant correlation with serum LDL-C levels. The g1679C > G SNP is located in exon 2 of the *NPC1L1* gene and is embedded within the coding region. This SNP was in linkage disequilibrium with known functional variants, such as *NPC1L1* -762T > C, an important transcription factor binding site, and −18C > A, in close proximity to a sterol regulatory element, thus suggesting that this SNP is related to the transcriptional level of *NPC1L1*; these changes lead to changes in the functionality of the NPC1L1 protein, thus leading to changes in the absorption of cholesterol (Tremblay et al., 2011).

When treated by monotherapy, patients with the CC genotype of the g1679C > G SNP tended to show significantly greater reductions in mean LDL-C (−23.99%) than either the GC genotype (−13.02% for pooled treatment groups; *p* < 0.01 for differences between pools) or the GG genotype (−16.45%, *p* < 0.01 for differences between pools) in patients who were treated with hyzetimibe (20 mg/day). When the effects of the placebo were removed, the reduction in LDL-C levels exhibited a rank order of CC (−20.49%) < GG (14.67%) < GC (−8.46%). The absolute reduction was 0.52 mmol/L for the GC genotype; this was compared to 0.65 mmol/L for the GG genotype and 0.96 mmol/L for the CC genotype.

Meta-analysis demonstrated that an absolute reduction of 1 mmol/L by statin treatment is associated with a 22% relative risk reduction in the 5-year incidence of major coronary events, ischemic stroke, and revascularization (Law et al., 2003). Most minimum doses for statins can result in an absolute reduction by more than 1 mmol/L and 0.73 mmol/L for ezetimibe. The EWTOPIA 75 (Ezetimibe Lipid-Lowering Trial on Prevention of Atherosclerotic Cardiovascular Disease in 75 or Older) was the first study to describe the clinical benefits of ezetimibe monotherapy in individuals aged ≥75 years with elevated levels of low-density lipoprotein cholesterol. In this trial, ezetimibe monotherapy was found to reduce the risks of the primary outcome by 34% (Ouchi et al., 2019).

Hence, patients with the heterozygous genotype (the GC genotype) should not be treated by hyzetimibe monotherapy due to poor efficacy. Patients with a homozygous genotype (the CC and GG genotypes) exhibited reductions of LDL-C [0.77 mmol/L (−19.34%)] similar to those of ezetimibe; in particular, the CC genotype exhibited significantly greater reductions in LDL-C than ezetimibe, thus indicating improved clinical benefits.

In contrast, and to our surprise, when we combined hyzetimibe and atorvastatin, we found that homozygous carriers (CC or GG) did not show a favorable dose response with regards to a further reduction of LDL-C (a 7.94% increase for CC and a 6.33% increase for GG); this was compared to a 15.29% increase for GC. Patients carrying the GC genotype showed a greater median reduction in LDL-C than those with the non-GC genotype (−52.23% *versus* −45.03%) in the hyzetimibe (20 mg) plus atorvastatin group (Figure 6). Furthermore, the proportion of patients exhibiting a reduction in the LDL-C level by >50% increased with increasing doses of hyzetimibe in the following rank order: 16.1% for atorvastatin alone, 46.2% for atorvastatin plus hyzetimibe (10 mg), and 65.4% for atorvastatin plus hyzetimibe (20 mg). These data showed that there was no feedback regulation induced by atorvastatin on the absorption of cholesterol in patients with the GC genotype.

Although there was a significant difference in the genotype frequency when compared between Caucasian, Chinese, and Japanese patients with hyperlipidemia, the frequency of the GC genotype in patients with the g1679C > G SNP ranges from 40 to 50%, thus indicating an abundance of individuals who would not respond to ezetimibe or hyzetimibe monotherapy. Therefore, it would be very important to remove such patients from such therapies due to their weak response to cholesterol absorption inhibitors and their poor effect on LDL-C reduction. It is important to consider that patients with a high risk of arteriosclerotic cardiovascular disease (ASCVD) need to control their cholesterol levels efficiently if they are to reduce the risk of cardiovascular events, thus demanding an appropriate lipid-lowering regimen from the beginning of treatment but not to modify according to the efficacy obtained after at least one month.

With regards to combination therapy, a previous drug–drug interaction study of atorvastatin and hyzetimibe showed there was no interaction between these drugs; this was also the case for the GC genotype. Thus, when the dose of atorvastatin or hyzetimibe increases, we should see a greater reduction in LDL-C.

By deploying an appropriate study design, our study found that variations in the *NPC1L1* gene had differential impacts on the efficacy of cholesterol absorption inhibitors; researchers have been attempting to address unravel these effects for the past 15 years. This is the first study to identify common SNPs and genotypes of the *NPC1L1* gene with respect to LDL-C reduction by more than 6%. The inconsistencies of previous studies relating to genotype analysis can be explained by differences in genotype frequency distribution between Eastern and Western ethnic groups and differences in the extent of genotype feedback regulation when different drugs are combined. These results are expected to provide significant guidance for the development of new inhibitors of cholesterol absorption with better efficacy, the design of corresponding clinical protocols, and the optimization of clinical hyperlipidemia treatments.

This study has several important limitations that need to be considered. First, the g1679C > G SNP in the *NPC1L1* gene exerts significant impact on LDL-C reduction during hyzetimibe treatment. This is because the chemical structure of hyzetimibe is different from that of ezetimibe. Furthermore, the affinities of these drugs to the NPC1L1 protein also differ; the precise impact of the *NPC1L1* gene on the action of ezetimibe is yet to be investigated. Second, subjects with the CC genotype achieved levels of LDL-C that were two-fold lower in response to hyzetimibe monotherapy treatment than subjects with the GC genotype (−23.99% *versus* −13.02%). The extent of LDL-C reduction showed a linear relationship with the dose of hyzetimibe. However, these data were obtained from a small number of patients; future research should involve a larger sample size if we are to fully validate our findings. Third, the frequency of the GG, GC, and CC genotypes was 41%, 46%, and 13% in Chinese patients with hypercholesterolemia; this compares to 5%, 40%, and 55% in Canadian patients with hypercholesterolemia (*p* < 0.001), respectively. The prevalence of the G allele variants indicates that the g1679C > G SNP exhibits ethnic specificity. However, the effects of hyzetimibe treatment on Caucasian patients with hypercholesterolemia are yet to be fully elucidated. Finally, we identified the notable impact of the 1735 C > G SNP on serum LDL-C levels in response to hyzetimibe treatment; however, we still need to identify the effects of a greater dose of hyzetimibe monotherapy in patients with the CC genotype. In addition, it would be very interesting to investigate whether higher doses of hyzetimibe or atorvastatin (in combination with each other) could achieve a better cholesterol-lowering target.

Dr. Martin Bdtker Mortensen and Brge Grnne Nordestgaard recently reported the outcomes of the Copenhagen General Population Study (CGPS) in The Lancet (2020) (Mortensen and Nordestgaard, 2020). Individuals aged 80–100 years with an LDL cholesterol level of 5.0 mmol/L or higher had the highest absolute risk of myocardial infarction and atherosclerotic cardiovascular disease than those aged 70–79 years [hazard ratio (HR): 2.99 *versus* 1.82], thus suggesting that active preventive strategies aimed at reducing the burden of myocardial infarction and atherosclerotic cardiovascular disease in the growing population aged 70–100 years should be adopted and may need more appropriate drugs to reduce the increased levels of LDL-C. Interestingly, at the same time and in the same journal, Gencer et al. also reported outcomes related to the efficacy and safety of lowering LDL cholesterol in older patients aged ≥75 years. This analysis involved 21,492 individuals from 29 trials; 54.7% were treated with statins, 28.9% were treated with ezetimibe, and 16.4% were treated with PCSK9 inhibitors. The risk of major vascular events in older patients was reduced by 26% per 1 mmol/L reduction in LDL-C; there was no significant difference in this respect when compared to patients aged <75 years. Furthermore, there were no significant differences between statin and non-statin treatments (Gencer et al., 2020). The clinical benefits of non-statin treatment (ezetimibe monotherapy) in individuals aged ≥75 years were also reported in the EWTOPIA 75 study (Ouchi et al., 2019). However, the HPS2-THRIVE (heart protection study 2-treatment of HDL to reduce the incidence of vascular events) study reported a rate of myopathy that was 10 times higher in Chinese patients receiving treatment with statins than that in European patients (HPS2-THRIVE Collaborative Group, 2013). In addition, in 2017, Wu et al. revealed that just 2.9% of high-intensity statins were used in China, thus resulting in an extremely low proportion of patients (<30%) of patients with CAD achieving a target LDL-C level (<1.8 mol/L) (Wu et al., 2017).

Therefore, ezetimibe or hyzetimibe monotherapy may be potentially useful for the treatment of older patients with dyslipidemia, especially those with statin-related adverse events (e.g., rhabdomyolysis) and in Chinese patients with hypercholesterolemia who are intolerant to high doses of statins.

The 2018 AHA/ACC treatment guidelines for LDL-C reduction recommended individualized treatment approaches for LDL-C reduction based on an individual patient’s CV risk category (Grundy et al., 2019). Cholesterol absorption inhibitors and PCSK9 inhibitors are the only drugs that are currently recommended as second-line treatment options as add-on therapies for patients with a high risk of CV who require additional LDL-C reduction. Patient access to PCSK9 inhibitors has been challenging due to expense and the refusal of payers to provide an appropriate cover. Inhibitors of cholesterol absorption remain as an optional choice with cheap expense; however, LDL-C reduction and the relative risk reduction of cholesterol absorption inhibitors are relatively small when compared to statins or PCSK9 inhibitors. Therefore, it appears to be important and of clinical significance to identify poor responders and non-responders and get rid of them for more effective interventions.

## Conclusion

Elevated cholesterol is an established risk factor for cardiovascular (CV) disease. Hyzetimibe represents a new inhibitor of cholesterol absorption and provides an additional therapeutic option for high-risk patients who cannot meet clinical goals with standard forms of therapy (maximally tolerated statin). The IMPROVE-IT trial first demonstrated that the coadministration of an inhibitor of cholesterol and a statin was associated with a lower incidence of cardiovascular events than statin monotherapy (Cannon et al., 2015; Giugliano et al., 2018). The EWTOPIA 75 trial further demonstrated the clinical benefits of monotherapy with cholesterol absorption inhibitors in individuals aged ≥75 years with elevated levels of low-density lipoprotein cholesterol (Ouchi et al., 2019). However, the modest treatment effects of hyzetimibe on LDL-C levels and the significant variability in response to this drug demands a comprehensive and targeted study of gene variation and the effects of such variation on drug response. The g1679C > G SNP in the *NPC1L1* gene is critical and can exert notably different impacts on the response to hyzetimibe treatment. Patients who are heterozygous (the GC genotype) will exhibit poor efficacy with hyzetimibe monotherapy. However, when co-administered with atorvastatin, patients with heterozygous genotypes achieved much better improvements in LDL-C levels. The results of our trial indicate that the precise treatment of hypercholesterolemia with hyzetimibe would be beneficial for patients and highlights the potential necessity for identifying the most appropriate subjects for treatment with ezetimibe.

## Data Availability

The original contributions presented in the study are publicly available. This data can be found here: https://www.ebi.ac.uk/eva/?Home, PRJEB49213.
