# Supplementary material for: The NPC1L1 Gene Exerts a Notable Impact on the Reduction of Low-Density Lipoprotein Cholesterol in Response to Hyzetimibe: A Factorial-Designed Clinical Trial
Source: Front Pharmacol. 2022 Mar 11;13:755469. doi: 10.3389/fphar.2022.755469 (PMC8963242; doi:10.3389/fphar.2022.755469)
Supplement: Supplementary file 1 [file Table8.DOC]

**Supplementary Table S2. Changes of LDL-C at the study time points compared to the baseline value**

| Time point | Placebo | ATO | HS25-10mg | ATO+HS25-10mg | HS25-20mg | ATO+HS25-20mg |
| --- | --- | --- | --- | --- | --- | --- |
|  | (n=88, %) | (n=70, %) | (n=74, %) | (n=73, %) | (n=75, %) | (n=64, %) |
| 2w | -0.37±10.61 | -36.33±10.68 | -12.88±9.68 | -45.92±10.50 | -15.56±10.93 | -46.78±12.77 |
| 4w | -2.33±10.68 | -38.33±11.52 | -12.25±12.29 | -46.75±11.34 | -15.57±10.55 | -48.77±16.34 |
| 8w | -0.76±12.49 | -37.78±10.88 | -13.36±11.55 | -46.56±11.96 | -15.95±10.93 | -47.05±16.05 |
| 12w | -3.12±14.13 | -37.50±13.19 | -12.82±12.68 | -48.38±10.39 | -16.29±10.73 | -47.96±14.34 |

Values are mean±SD

Reduction rate of LDL-C= (LDL-C in every visit week - LDL-C in baseline)/ LDL-C in baseline
