# Supplementary material for: The NPC1L1 Gene Exerts a Notable Impact on the Reduction of Low-Density Lipoprotein Cholesterol in Response to Hyzetimibe: A Factorial-Designed Clinical Trial
Source: Front Pharmacol. 2022 Mar 11;13:755469. doi: 10.3389/fphar.2022.755469 (PMC8963242; doi:10.3389/fphar.2022.755469)
Supplement: Supplementary file 2 [file Table7.DOC]

**Supplementary Table S2. Demographic data of the analysis set**

| Group | Placebo  (n=88) | ATO  (n=70) | HS25-10mg  (n=74) | ATO + HS25-10mg  (n=73) | HS25-20mg  (n=75) | ATO + HS25-20mg  (n=64) | *P* value |
| --- | --- | --- | --- | --- | --- | --- | --- |
| Age (mean±SD) | 55.10±8.73 | 53.77±9.58 | 56.3±8.7 | 55.55±10.27 | 56.23±9.11 | 54.56±9.91 | 0.558 |
| Gender (male, %) | 36(40.91) | 31(44.29) | 25(33.78) | 25(34.25) | 27 (36.00) | 21(32.81) | 0.663 |
| *Han* nationality (%) | 85(96.59) | 67(95.71) | 71(95.95) | 68(93.15) | 72(96.00) | 61(95.31) | 0.938 |
| Height (cm, mean±SD) | 163.82±8.15 | 163.44±8.75 | 162.51±7.27 | 161.38±8.10 | 162.80±7.05 | 162.67±7.84 | 0.491 |
| Weight (kg, mean±SD) | 65.59±11.02 | 67.47±13.76 | 63.99±9.53 | 63.17±11.75 | 63.84±10.98 | 64.78±11.04 | 0.248 |
| BMI (kg/m^2^, mean±SD) | 24.35±2.99 | 25.05±3.30 | 24.18±2.83 | 24.12±3.11 | 23.98±3.15 | 24.35±2.67 | 0.356 |
| LDL-C  (mmol/L, mean±SD) | 3.93±0.38 | 4.00±0.35 | 3.96±0.32 | 4.00±0.37 | 3.99±0.38 | 3.99±0.37 | 0.793 |

BMI, body mass index

LDL-C, low-density lipoprotein.

Gender is presented as Frequency (Proportion). Other values are presented as Mean (SD) and Range
